# Thyroid Lymphoma as a Cause of Dysphagia and Dyspnea in a Patient without Palpable Nodules or Goiter

**DOI:** 10.1155/2009/385461

**Published:** 2009-10-15

**Authors:** Jarrod D. Frizzell, Brandon J. Perkins, R. Scott Morehead

**Affiliations:** ^1^Department of Internal Medicine, University of New Mexico School of Medicine, MSC10-5550, 1 University of New Mexico, Albuquerque, NM 87131, USA; ^2^Division of Pulmonary, Critical Care, and Sleep Medicine, Department of Internal Medicine, University of Kentucky College of Medicine, Kentucky Clinic L543, 740 South Limestone St, Lexington, KY 40536, USA

## Abstract

Tumors originating in the neck are well-known causes of progressive dysphagia and dyspnea (including stridor), and thyroid lymphoma is an uncommon example. Physical examination provides an important first step in the evaluation of such complaints, as tumors large enough to produce such symptoms are typically considered to be palpable, if not able to be seen grossly. In this case presentation, the authors describe a nonsubsternal thyroid lymphoma measuring 3 × 4 cm at its largest diameter, producing dysphagia and leading to respiratory emergency, that was entirely nonpalpable to physical exam even after confirmation of its presence by computed tomography.

## 1. Introduction

Thyroid lymphoma is an uncommon disease that should be considered in patients with an enlarging neck mass associated with dysphagia and hoarseness [1]. In a previously reported case series [2], the typical patient was an elderly female with painless thyroid enlargement, although compression of aerodigestive tract was common at presentation. Yet thyroid palpation has been shown to have poor sensitivity, detecting only 40% of nodules > 1.5 cm in comparison to ultrasonograpy [3]. We were unable to identify another report of thyroid lymphoma and respiratory failure due to tracheal compression in a patient with an unremarkable neck examination, particularly given that the mass was not substernal or mediastinal in location.

## 2. Case Presentation

A 78-year-old man with a history of emphysema, hypertension, and hypothyroidism presented to his primary care physician for routine followup. During the review of systems, he was found to have dyspnea on exertion reported as unchanged from his baseline as well as progressive dysphagia of solid foods over an unspecified period of time. Physical exam was significant only for central obesity and decreased breath sounds with prolonged expiration bilaterally. A modified barium swallow one week afterward showed mild esophageal dysmotility and small caliber of the esophagus throughout. During the exam, the technician made note of hoarseness, which the patient stated had been ongoing for three weeks.

Esophagogastroduodenoscopy was performed approximately six weeks following initial presentation. Note was made of difficultly passing the endoscope into the esophagus secondary to hypopharyngeal edema. Endoscopy was terminated prematurely due to respiratory distress and decreasing oxygen saturation.

Emergent intubation was performed and the patient was transferred to the intensive care unit. Approximately three hours postintubation, the patient was awake and communicative, and wished to be extubated. As he had been otherwise stable, the decision was made to extubate. Within minutes, he began having inspiratory stridor. He was able to explain that this “shortness of breath” had been increasingly experienced over the past few weeks, to the point when it was present at rest to varying degrees. The otolaryngology service was called to assist in evaluation of the airway and emergent intubation; examination was normal, including appearance and movement of the vocal cords, and the patient was reintubated successfully.

**Figure 1:**
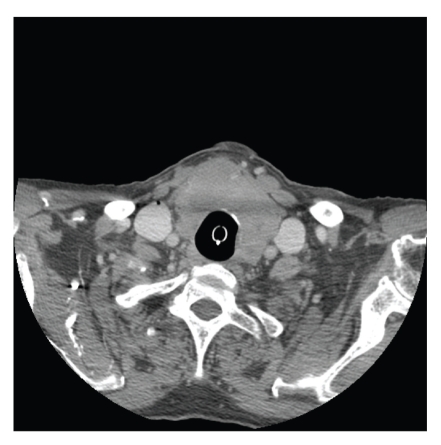
Thyroid mass at largest diameter of 3 × 4 cm.

**Figure 2:**
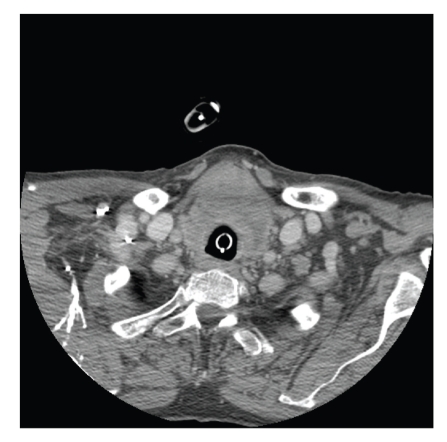
Thyroid mass at the level of the inflated endotracheal tube cuff; the cuff at this level is presumed to be stenting the tracheal airway surrounding the tube. Due to the emergent nature of the case, an extubated radiograph was unable to be obtained.

Palpation of the neck by the intensivist team as well as the surgical oncology service was significant for mild adiposity and loosened neck folds, but despite easy identification of anatomical landmarks, including the cricoid and apparent movement of the isthmus with swallowing, no goiter or nodules were palpable. Computed tomography (CT) of the neck was performed, which yielded a thyroid mass with compression of the trachea inferiorly (Figures 1 and 2). Even after imaging was obtained, no mass could be identified by palpation by any participating physician. CT-guided fine-needle aspiration of the mass was performed, which was nondiagnostic but with atypical lymphocytes. The aspirate was deemed inadequate for use in flow cytometry. Rather than attempt a second needle biopsy, open biopsy was chosen with the advantage of surgical decompression, and the mass was identified as diffuse large B-cell non-Hodgkin's lymphoma, positive immunostaining for CD45, CD10, and CD20.

## 3. Discussion

The trajectory of this patient's illness was unfortunate in many respects. The implicit reliance on a normal initial physical exam of the neck is often not applicable in the evaluation of elderly patients, as the thyroid exam in particular is more difficult in the elderly with either atrophy or increased substernal location playing a role [4]. Airway obstruction is a notorious complication of mediastinal goiters in particular, as the sternum provides an anterior boundary and the trachea is then subject to compression [5]. Notable in this case is that the patient's symptoms were not produced by a mediastinal or substernal mass; indeed, one would have thought based on imaging that its anterior cervical location would have rendered it easily palpable, which tragically was not the case. Though the patient's history of emphysema may have biased the perception of his dyspnea, in the setting of dysphagia this symptom should have warranted a more aggressive workup. Notation by the radiology technician of hoarseness should also have raised concern for pathology involving the upper airway, perhaps engendering a request for neck imaging. Given the insensitivity of physical examination, the history of progressive dysphagia combined with hoarseness and dyspnea should have dictated earlier evaluation of the airway prior to upper gastrointestinal endoscopy, possibly sparing the patient the emergency that transpired in this case.
